# Mate limitation and sex ratio evolution

**DOI:** 10.1098/rsos.171135

**Published:** 2018-02-07

**Authors:** Jussi Lehtonen, Lisa E. Schwanz

**Affiliations:** Evolution and Ecology Research Centre, School of Biological, Earth and Environmental Sciences, University of New South Wales, Sydney, New South Wales 2052, Australia

**Keywords:** sex ratio, sex allocation, local mate competition, fertilization, mate limitation, sperm limitation

## Abstract

Sex ratio evolution has been one of the most successful areas of evolutionary theory. Pioneered by Düsing and Fisher under panmixia, and later extended by Hamilton to cover local mate competition (LMC), these models often assume, either implicitly or explicitly, that all females are fertilized. Here, we examine the effects of relaxing this assumption, under both panmictic and LMC models with diploid genetics. We revisit the question of the mathematical relationship between sex ratio and probability of fertilization, and use these results to model sex ratio evolution under risk of incomplete fertilization. We find that (i) under panmixia, mate limitation has no effect on the evolutionarily stable strategy (ESS) sex allocation; (ii) under LMC, mate limitation can make sex allocation less female-biased than under complete fertilization; (iii) contrary to what is occasionally stated, a significant fraction of daughters can remain unfertilized at the ESS in LMC with mate limitation; (iv) with a commonly used mating function, the fraction of unfertilized daughters can be quite large, and (v) with more realistic fertilization functions, the deviation becomes smaller. The models are presented in three equivalent forms: individual selection, kin selection and group selection. This serves as an example of the equivalence of the methods, while each approach has their own advantages. We discuss possible extensions of the model to haplodiploidy.

## 1. Introduction

The theory of sex allocation has often been called one of the most successful areas of modern evolutionary theory. Pioneered by Düsing [1] and Fisher [2] for panmictic populations, and later extended by Hamilton [3] to populations with local mate competition (LMC), it has blossomed into an expansive combination of theory and data (reviewed in [4]). Despite extensive theoretical work, one aspect of the biological process that has received relatively little attention is that of possible mate limitation, and resulting incomplete fertilization of females. Often this assumption is not mentioned explicitly at all [2], while in other models there is a mathematical assumption that all females in a local mating group are fertilized, regardless of the number of males present [3]. The problem with this assumption is clearest in an LMC model in the case where there is just one foundress, and all matings take place between siblings [3]. The sex ratio predicted mathematically is such that no males are produced, and it is clear that no females could be fertilized under such conditions. A common verbal interpretation of this is that females are selected to produce the minimum number of males required to fertilize all of their daughters [3–6], and this is sometimes taken literally as a prediction of Hamilton's model.

**Figure 1.**
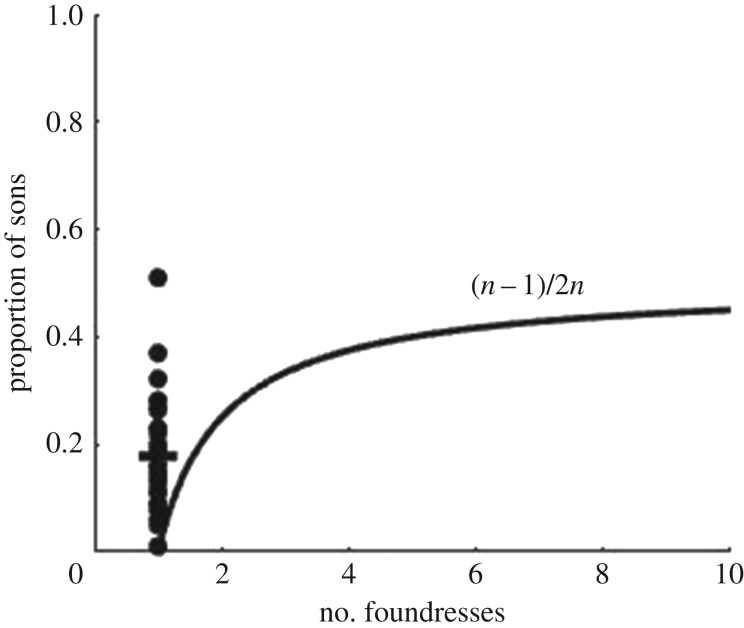
Under LMC, the predicted proportion of sons increases as foundress number increases [3] (curved line). Empirical data often depart strongly from this pattern for single foundresses (scatter; line = mean; see the electronic supplementary material). A high proportion of sons has been attributed to fertility insurance under male mortality, and to variation in clutch size (reviewed in [4]), assuming one son surviving to mating age is sufficient to fertilize all daughters. Here, we relax the last assumption. Note that the figure is based on data from haplodiploid and pseudo-arrhenotokous species (see the electronic supplementary material), and hence does not match the assumption of diploidy in our model. However, the predicted evolutionarily stable strategy (ESS) sex allocation for single foundresses is 0 for both diploid and haplodiploid models under LMC when mate limitation is not accounted for [4], and the data clearly show a deviation from this simple mathematical prediction.

Empirically, the proportion of sons produced by a single foundress in an LMC scenario is often quite large (figure 1), raising questions about the effect of mate limitation in sex ratio evolution. Previous theory has examined the effects of clutch-size variation and of the risk that all male fertilizers die before mating [7–9] (reviewed in [4]). These models still assume that a single male is sufficient to fertilize any number of females, which implies that they cannot be used to explicitly examine the verbal interpretation mentioned above. The models are also generally restricted to small numbers of foundresses.

We clarify and extend previous sex allocation theory [1–3] by explicitly modelling insemination limitation in males, so that one male is no longer necessarily sufficient to fertilize all females. If the fertilization limit is set to infinity, it matches the assumption that one male is capable of fertilizing any number of females. We use a mating function that has been used in previous work on mate limitation [10] and derive two novel mating functions based on explicit biological considerations. The simple verbal LMC prediction (the evolutionarily stable strategy (ESS) sex ratio is always such that all females can be fertilized) is only borne out with one of these functions, while the other two can lead to significant mate limitation at the ESS, in contrast to the usual interpretation of LMC models with one foundress.

The model is analysed from individual selection, kin selection and group selection viewpoints, where the last two allow an analysis of the panmictic ‘Fisherian’ scenario as a special case. These different interpretations are all equivalent in terms of the evolutionary outcome, but it is nevertheless useful to present all three: the importance of, e.g. group selection in sex ratio evolution has been much debated [11,12], and researchers with different methodological preferences may choose to focus on a different version of the model, which can be thought of as different causal interpretations of the evolutionary process [13]. Furthermore, the three interpretations of the model serve as an illustration of the mathematical equivalence of the individual, kin and group selection methods.

## 2. Models

### 2.1. A simple, motivational example

We begin with a simple LMC example that demonstrates two important points: (i) that mate limitation can influence the stable sex ratio, increasing the proportion of males and (ii) despite the increased number of sons, the stable sex ratio does not necessarily ensure the fertilization of all daughters. We address this with the simple case of LMC when there is one foundress, where 100% investment into females is predicted by early models [1].

Notation for all models is given in table 1. In the simple situation of just one foundress per group, the average sex allocation by foundresses in the group (*z*) necessarily equals that of the focal foundress (*x*). Hence, she produces a total of *k*(1 − *x*) female offspring, with average proportion *f*(*z*) = *f*(*x*) of the daughters' eggs fertilized. These fertilized eggs are all sired by sons of the same sole foundress, which implies that every fertilized egg can be thought to yield identical fitness benefits to the foundress via sons and daughters, giving total fitness *k*(1 − *x*) *f*(*x*) +* k*(1 − *x*) *f*(*x*), proportional to

2.1 w=(1−x)f(x)+(1−x)f(x).

We retain the two identical terms (arising through sons and daughters) separately in equation (2.1) to facilitate comparison with the more general equation (2.3) below.

**Table 1.** Notation and parameters.

notation	name of parameter, variable or equation
*x*	proportion of male offspring produced by the focal foundress in the ESS models: males/(males + females)
*y*	proportion of male offspring produced by non-focal foundresses in the ESS models
*z*	average proportion of male offspring produced by a group of foundresses
x*	ESS value of the proportion of male offspring
*f*(*z*)	proportion of fertilized females as a function of group average sex ratio
*q*	group mean ratio of males to females: *q *= *z*/(1 − *z*)
*n*	number of foundresses per patch
*k*	number of offspring per foundress
*a*	mating parameter. In *f*_2_ and *f*_3_ (table 2), *a* is the maximum number of matings a single male is capable of. In *f*_1_, it has a similar overall effect, but has no obvious biological interpretation.

Because there is just one foundress, and hence *x *= *z*, the model reduces to a simple frequency-independent optimization problem where there is a trade-off between investment into females (1 − *x*) and the fertilization probability of those females *f*(*x*). This already makes it clear that there is no *a priori* reason why the optimum should be such that *f*(*x*) = 1. Instead, the optimum will depend on how fertilization success relates to the sex ratio. Consider the simple saturating function

2.2 $$f(x)=\frac{ax}{ax+1},$$

similar to that used in some previous models of mate limitation [10]. The positive parameter *a* determines the ‘efficiency’ of the fertilization process. To find the sex ratio that maximizes fitness, we plug this fertilization function into equation (2.1), differentiate and set the derivative to zero: $a(1-2x-ax^{2})\text{/}{\left(1+ax\right)}^{2}=0$. Solving this yields the optimum $x=(-1+\sqrt{1+a})\text{/}a$. The probability of fertilization at the ESS sex ratio is then $f\left((-1+\sqrt{1+a})\text{/}a\right)=1-\frac{1}{\sqrt{1+a}}=1-\sqrt{1+a}\text{/}(1+a)$. This is smaller than 1 for any finite value of *a* and can be arbitrarily close to 0 with small *a*. Therefore, it is incorrect to assume that selection will always ensure that all females are fertilized under LMC, and the effect deserves more detailed analysis.

### 2.2. A general model framework for local mate competition, local mate limitation and Fisherian sex ratio evolution

We now construct a model of sex ratio evolution that can be used to examine both the Hamiltonian (LMC) and Fisherian (panmictic) scenarios, and allows for simultaneous LMC and local mate limitation. We denote the proportion of males produced by the focal foundress by *x* and the group average (including the focal) sex ratio by *z*. In the panmictic case, where there is no division into groups (or alternatively, we can say there is only one group), *z* refers to the average sex ratio of the entire population. From this simple starting point, the model can be analysed using individual selection, kin selection and group selection.

#### 2.2.1. Fitness function

If there are *n* mothers, the proportion of mates gained by the sons of the focal mother is *x*/*nz*. In a Fisherian (panmictic) model, *n* refers to the entire population (i.e. the size of the sole group), and in an LMC model to the size of a local group with *n* foundresses. The total number of daughters by the *n* mothers is *nk*(1 − *z*), each with fertilization probability *f*(*z*). The fitness gained by the focal mother via sons is (x/nz)nk(1−z)f(z)=(x/z)k(1−z)f(z) and via daughters *k*(1 − *x*)*f*(*z*). Mating among the offspring in the group is assumed to be random, so that the fertilization probability *f*(*z*) depends only on the group mean sex ratio. Under the mating functions we consider, a female is either completely fertilized, or not at all, and *k* is a constant. Combining fitness via sons and daughters, and omitting *k* yields

2.3 $$w=\frac{x}{z}(1-z)f(z)+(1-x)f(z).$$

Note that if there is just one foundress, *z *= *x*, and (2.3) simplifies to (2.1).

#### 2.2.2. Individual selection

In an LMC model, the focal female may form a large proportion of her social group, and hence, her trait value (*x*) may have a large effect on the mean trait value in the group (*z*). We therefore denote the proportion of males produced by *non*-*focal* group members by *y*, and thus, z=(x+(n−1)y)/n. Combining this with equation (2.3), we can find the candidate ESS sex allocation by evaluating the derivative $\text{d}w\text{/d}x{|}_{x=y=x^{*}}$ and solving for the value of x*, where this derivative evaluates to zero [14]. Differentiating and simplifying, we obtain the general equation as follows:

2.4 $${\frac{\text{d}w}{\text{d}x}|}_{x=y=x^{*}}=\frac{\left(n-1-2nx^{*})f(x^{*})+2(1-x^{*})xf^{\prime }(x^{*}\right)}{nx^{*}}=0.$$

#### 2.2.3. Kin selection

Equation (2.3) can also be used as a starting point for a kin selection model. Using the ‘direct fitness’ approach [15–17], costs (−*c*) and benefits (*b*) are found by differentiating the fitness function for focal and group trait values *x* and *z*, respectively, and evaluating these at *x *= *z *= x*. We find that $-c=\partial w\text{/}\partial x{|}_{x=z=x^{*}}=(1-2x^{*})f(x^{*})\text{/}x^{*}$ and $b=\partial w\text{/}\partial z{|}_{x=z=x^{*}}=(2x^{*}(1-x^{*})f^{\prime }(x^{*})-f(x^{*}))\text{/}x^{*}$. Because *z* is the average sex ratio for the whole group that the focal mother interacts with, including her own value *x*, *R* is a whole-group relatedness coefficient [18,19]. Putting these components together, we have a kin selection model for finding a candidate ESS:

2.5 $$-c+bR=\frac{\left(1-2x^{*})f(x^{*}\right)}{x^{*}}+R\frac{2x^{*}(1-x^{*})f^{\prime }(x^{*})-f(x^{*})}{x^{*}}=0.$$

#### 2.2.4. Group selection

The model can be also interpreted in George Price's group selection formalism [20,21]. Although mathematically equivalent, kin selection and group selection (or multi-level selection) are not just alternative methods for addressing an evolutionary question—they also provide different causal interpretations of evolution [13]. Here, we take advantage of the fact that the *b*, *c* and *R* components of a kin selection model (equation (2.5)) can be rearranged to obtain the between- and within-group components of a group selection model as follows [22]:

2.6 $$\underset{\mathrm{between}\text{-}\mathrm{group}}{\underbrace{(b-c)R}}\;\underset{\mathrm{within}\text{-}\mathrm{grou}p}{\underbrace{-c(1-R)}}.$$

Substituting *b*, *c* and *R* from equation (2.5) into equation (2.6), the model can be recast as a group selection model:

2.7 $$\underset{\mathrm{between}\text{-}\mathrm{group}}{\underbrace{2[(1-x^{*})f^{\prime }(x^{*})-f(x^{*})]R}}+\underset{\mathrm{within}\text{-}\mathrm{group}}{\underbrace{\frac{\left(1-2x^{*})f(x^{*})(1-R\right)}{x^{*}}}}=0.$$

While the kin selection model (equation (2.5)) splits selection into the costs and benefits of Hamilton's rule [23,24] weighted by relatedness, the group selection model components represent selection that takes place between groups (due to differences in group fitness) and within groups (due to intra-group differences in individual fitness). We examine the group selection decomposition in further detail in the discussion.

#### 2.2.5. Whole-group relatedness *R*

The kin selection and group selection formulations of the model (equations (2.5) and (2.7)) contain a whole-group relatedness coefficient *R*. In an LMC model, we can think of *R* as average relatedness of a mother to all mothers in her patch, including herself. We do not need to separately consider a mother's relatedness to her sons and daughters, because in a diploid model a mother is equally related to sons and daughters [17]. In a Fisherian model, we think of *R* simply as the average relatedness of a mother to all mothers in the global population, including herself. Therefore, for specific results, we have two values of *R*. The simplest case is *R *= 0, which corresponds to Fisherian sex ratio evolution; the average relatedness (in a kin selection sense) to the entire population including self is 0, regardless of population size. For the Fisherian case, we could have, in fact, done without the direct fitness method (equation (2.5)) and used a standard ESS analysis, but including the relatedness coefficient explicitly in the model clarifies that the result is not dependent on population size. The second case that we analyse is Hamilton's [3] LMC scenario, where all females disperse after mating, and then give birth to their offspring in a new patch with a total of *n* foundresses. We further assume that there is a very large number of such patches. The focal female is hence unrelated to other, randomly dispersed foundresses in her patch, while her relatedness to herself is 1. Whole-group relatedness in our LMC models is therefore R=(1+(n−1)0)/n=1/n. It is easy to check that when this is plugged into equations (2.5) and (2.7), and the equations are simplified, they both become identical to equation (2.4).

### 2.3. The relation between sex ratio and probability of fertilization

Finding the ESS sex allocation using equation (2.4), (2.5) or (2.7) requires an explicit function *f*(*z*). The simplest example is *f*(*z*) = 1, meaning that all females are fertilized regardless of the sex ratio. Equation (2.4) then reduces to $(n-1-2nx^{*})=0$, which obtains Hamilton's [3] original ESS LMC solution $x^{*}=(n-1)\text{/}2n$.

We consider three different functions, shown in table 2. The first is a common, but phenomenological, model not derived from specific mechanistic theory f(z)=az/(az+1) [10]. To complement this approach, and to check for robustness of the results, we derive alternative functions, grounded in specific biological assumptions. Because these functions are primarily intended for a model of LMC where potential mates are usually confined in a relatively small space, we assume that the limiting factor is not difficulty in searching for mates, but rather limitations in how many females one male is able to fertilize in general. In a Fisherian model, we may well imagine scenarios where the opposite is true, but as we will see, the exact mathematical form of the function becomes irrelevant in the Fisherian case. Detailed derivations can be found in the electronic supplementary material. Note that *f*_2_ and *f*_3_ are functions of the ratio of males to females (q=z/(1−z)), and only indirect functions of the proportion of male offspring (*z*) as is typically used as a measure of sex allocation. This relationship arises naturally from the derivation (electronic supplementary material), and it may have implications for selection because *q* is a concave (accelerating) function of *z*.

**Table 2.** Mating functions used in the model. See the electronic supplementary material, for derivations of *f*_2_(*z*) and *f*3(*z*). *q* = *z*/(1 − *z*) proportion of males to females in a local group.

notation	function	rationale and assumptions
*f*_1_(*z*)	*az*/(*az *+ 1)	a phenomenological model which has been used in previous research [10], but is not derived explicitly from theory
*f*_2_(*z*)	1 − e^−*aq*^	males and females can mate multiply, the total number of mating individuals in a patch is reasonably large and(i) mating is indiscriminate and random so that a male does not avoid remating with the same femaleor(ii) males do avoid remating with the same female, but a single female can be mated by multiple males, and there are several individuals of both sexes
		here, *a* is the number of females each male is capable of fertilizing
*f*_3_(*z*)	min(*aq*, 1)	males can mate multiply, with at most *a* females; females mate only once

Details and stability analysis [14,25] for the ESS calculations using these functions can be found in the electronic supplementary material.

## 3. Evolutionarily stable strategy results

Substituting *R *= 0 into equation (2.5) or (2.7) yields $(1-2x^{*})f(x^{*})\text{/}x^{*}=0$, with the well-known Fisherian ‘equal allocation’ result x* = 1/2. This implies that mate limitation has no effect on the stable sex ratio under panmixia, regardless of the shape of the function *f*, even in populations that are so sparse that the vast majority of both sexes never find a mate (i.e. *f*(1/2) is close to 0).

**Figure 2.**
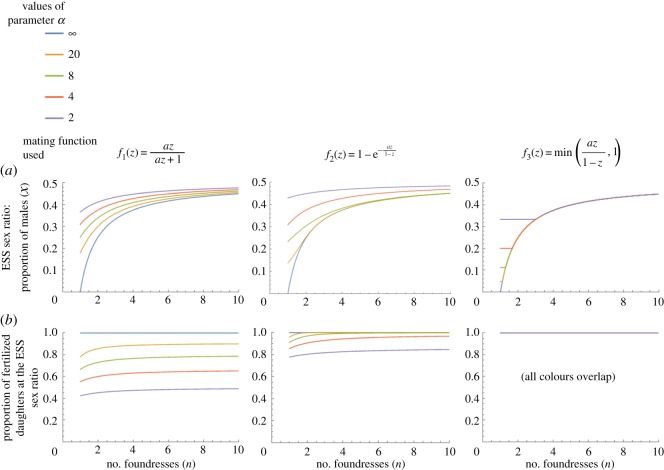
ESS sex ratio (*a*) and proportion of fertilized daughters (*b*). (*a*) How limited insemination capacity (*a* < ∞) makes the sex ratio deviates from the classic LMC prediction $x^{*}=(n-1)\text{/}2n$, while *a* = ∞ reproduces this result. Contrary to verbal predictions, the ESS sex ratio does not necessarily lead to 100% fertilization. The result depends strongly on the mating function. *f*_1_ is a common phenomenological model and leads to significant mate limitation at the ESS. *f*_2_ is based on the assumption that both sexes can mate multiply, while *f*_3_ assumes that females mate once and males multiply given the opportunity. Only *f*_3_ agrees with the verbal prediction that all females should be fertilized at the ESS sex ratio.

Under LMC, we find that mate limitation can have a significant effect on sex allocation, increasing allocation to male offspring compared with the baseline result of LMC with no mate limitation [3]. However, there is no guarantee that enough males are produced at the ESS to fertilize all daughters. ESS sex allocation results for all three mating functions (table 2) are shown in figure 2. Substituting the first function $f_{1}(z)=az\text{/}(az+1)$ into equation (2.4) (or alternatively, into equation (2.5) or (2.7) with *R *= 1/*n*) and solving for the ESS yields

3.1 $$x^{*}=\frac{a(n-1)-2(n+1)+\sqrt{8an(n+1)+{\left[a(n-1)-2(n+1)\right]}^{2}}}{4an}$$

With the function $f_{2}(z)=1-{\text{e}}^{-az\text{/}(1-z)}$, the ESS must be computed numerically. Applying this function to equation (2.4) and simplifying obtains

3.2 $$\frac{{\text{e}}^{ax^{*}\text{/}(-1+x^{*})}(-1+n+x^{*}-2ax^{*}-3nx^{*}+2n{x^{*}}^{2})-(-1+x^{*})(1+n(-1+2x^{*}))}{n(-1+x^{*})x^{*}}=0,$$

from which x* can be solved for any combination of *a* and *n*.

$f_{3}(z)=\min(az\text{/}(1-z),\;1)$ again allows for an explicit solution. Because of the piecewise definition of *f*_3_(*z*), the ESS solution will also be piecewise. A detailed solution can be found in the electronic supplementary material. Assuming that *a *≥ 1 (a single male is capable of inseminating at least one female, as is likely in nature), the ESS is

3.3 $$x^{*}=\max\left(\frac{n-1}{2n},\frac{1}{1+a}\right)\;(a\geq 1).$$

In all three cases, the optimal proportion of sons declines as the number of foundresses declines (figure 2). However, we find that more sons are produced when there are limitations on insemination (a<∞) than when there are no limitations, particularly for single foundresses.

We can also find the proportion of fertilized females at the ESS by plugging these ESS values back into the corresponding mating functions (figure 2). For most parameter values in the first two functions, we find that less than 100% of daughters are fertilized at the ESS sex ratio, with an exacerbated effect for single foundresses. For *f*_3_, we find that $f_{3}(x^{*})=1$. In other words, with *f*_3_, the verbal prediction is borne out: the ESS is always such that all daughters are fertilized.

## 4. Discussion

Here, we have examined the effect of mate limitation on sex ratio evolution in Fisherian (panmictic) and Hamiltonian (LMC) models. The Fisherian case confirms that under panmixia, mate limitation has no effect on the stable sex ratio, no matter how severe. Intuitively, while a mutant that produces more males may relieve mate limitation in the offspring generation, the benefit is bestowed upon random members of the population, whose genotype is a random sample of the population genotype. Hence, reduction in mate limitation has no effect on gene frequencies in the population. In LMC models, in contrast, we find that mate limitation has a significant effect on stable sex ratios, increasing allocation to male offspring. However, our results indicate that it is not necessarily true that selection under LMC leads to sex ratios where all females are fertilized even when there is no offspring mortality. Instead, the outcome depends on the mathematical relationship between the sex ratio and fertilization probability. Realistic mathematical relationships can lead to significant mate limitation at the ESS (figure 2).

There are some important differences between the results from the different mating functions. The extent of mate limitation at the ESS decreases from *f*_1_ to *f*_3_ (figure 2*b*). With *f*_1_, a large fraction of females can remain unfertilized at the ESS sex ratio, contrary to the verbal prediction of previous LMC models. On the other hand, *f*_1_ is probably the least realistic of the three functions. For example, even when the population is nearly all male, fertilization probability approaches $f_{1}(1)=a\text{/}(a+1)$, which is only close to 100% fertilization with very large values of *a*. Including more realistic functions derived explicitly from biological considerations shows that the expected mate limitation at the ESS is probably less drastic than the results with *f*_1_ would suggest. At the opposite extreme, fertilization probability at the ESS with *f*_3_ is always 100%, in line with the verbal prediction. The results with function *f*_2_ lie between these extremes. The main biological difference between *f*_2_ and *f*_3_ is that in the former, females can mate multiply, while in the latter they never mate more than once. It is also interesting to note that these results are not constrained to the case of a single foundress, and mate limitation can extend to multiple foundress scenarios (figure 2*b*).

### 4.1. Alternative model interpretations

We have presented models of the same biological process under individual selection, kin selection and group selection guises, and this has several advantages. One general advantage is simply that these methods have been the source of much disagreement in recent years [26], and presenting all three may pre-empt such disagreements in the context of the current model. There are also more specific advantages to each approach. Checking for convergence and evolutionary stability [14,25] is easiest using the individual selection approach (equation 2.4; see electronic supplementary material, for stability analysis). The kin selection approach (equation (2.5)) connects the current work to previous significant discussions on sex ratio evolution using kin selection methods (e.g. [17]) and serves as a potential starting point for a future haplodiploid model (see below). The group selection interpretation (equation (2.7)) provides an interesting alternative causal interpretation of the model. The between-group and within-group components are $2[(1-x^{*})f^{\prime }(x^{*})-f(x^{*})]R$ and $\left(1-2x^{*})f(x^{*})\text{/}x^{*}(1-R\right)$, respectively. The between-group component has a clear causal interpretation: group fitness is increased if fertilization probability is increased, or if more females are produced. The former happens when male allocation is increased $\left(\;f^{\prime }(x^{*})\right)$, and the latter when male allocation is decreased, at a rate proportional to the current fertilization probability of females $\left(-f(x^{*})\right)$. These two components of between-group selection are therefore opposed, where one selects for increased male allocation, and the other for increased female allocation. But for the between-group component to have any effect, there must be grouping which leads to *R *> 0. Without positive *R*, all that remains is the within-group component which gives the standard Fisherian 50 : 50 sex allocation result, regardless of the severity of mate limitation.

### 4.2. Diploidy and haplodiploidy

The model presented here is a diploid model, in the sense that it does not account for the effect that inbreeding can have on sex ratio evolution in haplodiploids [4,27], nor for their sex determination system, where unfertilized eggs develop into males [28]. Many species experiencing LMC are haplodiploid, raising the question as to whether mate limitation is less of a concern for those species. The answer to this question is not obvious, for several reasons. Firstly, fitness from unfertilized eggs is not necessarily lost entirely, because they can still develop as males. Second, haplodiploid females might lose control of their offspring sex ratio to some extent due to suboptimal fertilization. Third, the fitness value of these extra males would depend on both the available fertilization opportunities (dependent on the number of foundresses) and the extent of mate limitation (also potentially indirectly dependent on the number of foundresses; figure 2). Fourth, the realized sex ratio in a local group or in the population is not directly determined by the sex ratio strategy of mothers. Unfertilized females give birth to males, inflating average male allocation. Therefore, there seems to be a recursive feedback between the sex ratio strategy, the proportion of fertilized females and the sex ratio in the next generation. For these reasons, a haplodiploid model would be significantly more complex than the diploid models analysed here.

However, an alternative interpretation of the diploid models, combined with a kin selection approach, suggests a way forward with a haplodiploid model: one approach to sex ratio modelling is to consider the division of reproductive value in the population [2,17]. In Fisherian models, total reproductive value of each sex is split evenly between all offspring of that sex. In Hamilton's LMC model [3], reproductive value is still split evenly between female offspring, but not necessarily between male offspring. By diverting resources towards females, a mutant mother can increase the *per capita* reproductive value of every male offspring in her patch, including her own sons. This happens for two reasons: for the son of a mutant mother, a smaller number of brothers means less competition for matings, and a larger number of sisters means more mating partners [29]. The effect of mate limitation, on the other hand, is to decrease the reproductive value of both sons and daughters. All else being equal, both sons and daughters in a less mate-limited patch have an inflated reproductive value compared with a more mate-limited patch. This reproductive value-based approach combined with kin selection methodology would be a natural way to extend the model to haplodiploidy. In diploids, mother–son relatedness equals mother–daughter relatedness, and total reproductive value of juvenile males equals that of juvenile females. Neither of these is true in haplodiploids [17]. Hence, a careful accounting of relatedness and reproductive value of sons and daughters is necessary in haplodiploid models anyway, and it would provide a way to consider the additional effect of mate limitation on the division of reproductive value in the population under haplodiploid sex determination. We leave the haplodiploid problem for future work.

## Supplementary Material

Supplementary material for ‘Mate limitation and sex ratio evolution’: Data for Fig. 1, and additional mathematical derivations.

## Data accessibility

The electronic supplementary material contains the data for figure 1 and additional mathematical derivations.
