# Supplementary material for: Mate limitation and sex ratio evolution
Source: R Soc Open Sci. 2018 Feb 7;5(2):171135. doi: 10.1098/rsos.171135 (PMC5830725; doi:10.1098/rsos.171135)
Supplement: Supplementary material for ‘Mate limitation and sex ratio evolution’: Data for Fig. 1, and additional mathematical derivations. [file rsos171135supp1.pdf]

Supplementary material for

Mate limitation and sex ratio evolution

Jussi Lehtonen and Lisa Schwanz

Evolution and Ecology Research Centre, School of Biological, Earth and Environmental Sciences, University of New South Wales, Sydney, New South Wales 2052, Australia.

Corresponding author: Lehtonen, J. (jussi.lehtonen@iki.fi)

1) Data for Fig. 1: Empirical sex ratios for one foundress conditions

Table S1. Mean sex ratio (proportion male) produced by single foundresses. Data are from publications presented in West et al. (2005; Appendix) as focusing on the role of foundress number under Local Mate Competition. Ploidy: HD = haplodiploid; PA = Pseudo-arrhenotokous. When multiple treatments existed at single foundress conditions, the group sex ratio were averaged.

Reference	Species	Ploidy	Proportion male with 1 foundress
Cremer and Heinze 2002	*Cardiocondyla obscurior*	HD	0.265
Peer and Taborsky 2004	*Xylosandrus germanus*	HD	0.06
Borsa and Kjellberg 1996	*Hypothenemus hampei*	PA	0.08
Roeder et al. 1996	*Tetranychus urticae*	HD	0.264
Roeder 1992	*Tetranychus urticae*	HD	0.227
Nagelkerke and Sabelis 1998	*Typhlodromus occidentalis*	PA	0.322
Nagelkerke and Sabelis 1998	*Phytoseiulus persimilis*	PA	0.114
Moore et al. 2002	*Liporrhopalum tentacularis*	HD	0.11
Frank 1985	*Pegascapus assuetus*	HD	0.09
Herre 1985	*Pegascapus (Blastophaga) sp. 2*	HD	0.14
Herre 1985	*Pegascapus (Blastophaga) sp. 1*	HD	0.19
Herre 1985	*Tetrapus costaricensis*	HD	0.22
Sagarra et al. 2000	*Anagyrus kamali*	HD	
Jackson 1996	*Caraphractus cinctus*	HD	0.23
Abe et al. 2003	*Melittobia australica*	HD	0.01
Cooperband et al. 2003	*Melittobia digitata*	HD	0.05
King and Seidl 1993	*Muscidifurax raptor*	HD	0.37
King and Skinner 1991	*Nasonia giraulti*	HD	0.06
King and Skinner 1991	*Nasonia vitripennis*	HD	0.16

Werren 1980	*Nasonia vitripennis*	HD	0.087
Werren 1983	*Nasonia vitripennis*	HD	0.15
Shuker and West 2004	*Nasonia vitripennis*	HD	0.2
Molbo and Parker	*Nasonia vitripennis*	HD	0.112
Takagi 1986	*Pteromalus puparum*	HD	0.11
King 1989	*Spalangia cameroni*	HD	0.51
Rabinovich 2000	*Telenomus fariai*	HD	0.13
van Welzen and Waage 1987	*Telenomus remus*	HD	0.11
Schwartz and Gerling 1974	*Telenomus remus*	HD	0.271
Waage and Lane 1984	*Trichogramma evanescens*	HD	0.18
Salt 1937	*Trichogramma evanescens*	HD	0.224
Luck et al. 2001	*Trichogramma pretiosum*	HD	0.282

2) Derivation of the mating function $f_2(z) = 1 - e^{-a\frac{z}{1-z}} = 1 - e^{-aq}$ (table 2)

Assume that each male can successfully engage in a matings on a given patch, in the time available for matings. If males mate indiscriminately, so that each female can be mated multiple times, and a male does not avoid re-mating with the same female, then:

i) The total number of matings is αzl, where *l* is the number of total number of individuals in the patch (i.e. all the offspring of both sexes of all foundresses).

ii) Because there are $(1-z)l$ females, the probability of a given female being mated in a given mating event is $\frac{1}{(1-z)l}$, and the probability of not being mated in this event is $1 - \frac{1}{(1-z)l}$.

iii) The probability of a given female not being mated in any of the αzl matings is $\left(1 - \frac{1}{(1-z)l}\right)^{\alpha zl}$.

iv) If *l* is reasonably large, then $\left(1 - \frac{1}{(1-z)l}\right)^{\alpha zl} \approx \lim_{l\to\infty}\left(\left(1 - \frac{\frac{1}{(1-z)}}{l}\right)^{l}\right)^{\alpha z} = \left(e^{-\frac{1}{(1-z)}}\right)^{\alpha z} = e^{-\alpha\frac{z}{(1-z)}} = e^{-\alpha q}$ which follows from the limit definition of e. Note that $q = \frac{z}{1-z}$ =proportion of males to females.

v) Therefore, the probability of a given female being mated is approximately $1 - e^{-\alpha q}$.

vi) The same would follow from assuming that the number of matings per female is Poisson distributed with mean $\frac{\alpha zl}{(1-z)l} = \alpha\frac{z}{1-z} = \alpha q$. Using the Poisson distribution is similarly justified when *l* is reasonably large, because then a) the number of 'trials' (αzl), becomes large and b) the probability of success in each trial $\left(\frac{1}{(1-z)l}\right)$ becomes small.

vii) Assuming that *l* is large, the same function can be found with a similar derivation even if a given pair never mates twice, but both males and females can mate multiply.

3) Derivation of the mating function $f_3(z) = \min(aq, 1) = \min(a\frac{z}{1-z}, 1)$ (table 2)

Again assume that a single male is capable of a matings, but females only mate once, after which they leave the pool of available mates. Now the total number of potential matings alz may exceed the actual matings, being limited by the number of females $l(1-z)$. Therefore the number of matings is $\min[alz, l(1-z)]$, and the proportion of mated females is $\frac{\min[alz, l(1-z)]}{l(1-z)}$, so that

$$f(z) = \min(\frac{az}{1-z}, 1) \quad or \quad f(z) = \min(aq, 1), \text{ where } q = \frac{z}{1-z}$$

4) Stability analysis of equation (8)

To be an ESS (Eshel et al. 1997), the candidate trait value must satisfy the criterion

$$\left.\frac{d^2w}{dx^2}\right|_{x=y=x^*} < 0$$

and to be convergence stable (Eshel et al. 1997), the criterion

$$\left.\frac{d^2w}{dx^2}\right|_{x=y=x^*} + \left.\frac{d^2w}{dxdy}\right|_{x=y=x^*} < 0$$

With the first mating function we find after simplification

$$\left.\frac{d^2w}{dx^2}\right|_{x=y=x^*} = -\frac{2a}{n^2(1+ax^*)^3}[2n + (n+1)a + (n-1)x^*(a^2 + 2a)]$$

and

$$\left.\frac{d^2w}{dx^2}\right|_{x=y=x^*} + \left.\frac{d^2w}{dxdy}\right|_{x=y=x^*} = -\frac{a}{n(1+ax^*)^3}[2(n+1) + a(n+3) + (n-1)(a^2x^* + 2ax^*)]$$

In both cases it is easy to see that the components in square brackets are positive when $x^* \geq 0$, $a \geq 0$ and $n \geq 1$; therefore equation (8) is an ESS and convergence stable.

5) Stability analysis of equation (9)

With the mating function $f_2(x)$ we are restricted to numerical solutions for the equilibria. Therefore we also take a partly numerical approach to stability analysis.

First we derive

$$\left.\frac{d^2w}{dx^2}\right|_{x=y=x^*} =$$

$$-\frac{2e^{\frac{ax^*}{-1+x^*}}(-1+e^{\frac{ax^*}{1-x^*}}(-1+n)(-1+x^*)^3+3x^*-ax^*-3x^{*2}+3ax^{*2}-a^2x^{*2}+x^{*3}-2ax^{*3}+n(-1+x^*)(-1-(-2+a)x^*+(-1+2a)x^{*2}))}{n^2(-1+x^*)^3x^{*2}}$$

and

$$\left.\frac{d^2w}{dx^2}\right|_{x=y=x^*} + \left.\frac{d^2w}{dxdy}\right|_{x=y=x^*} =$$

$$-\frac{e^{\frac{ax^*}{-1+x^*}}(-1+e^{\frac{ax^*}{1-x^*}}(-1+n)(-1+x^*)^3+3x^*-ax^*-3x^{*2}+3ax^{*2}-2a^2x^{*2}+x^{*3}-2ax^{*3}+n(-1+x^*)(-1-(-2+a)x^*+(-1+2a)x^{*2}))}{n(-1+x^*)^3x^{*2}}$$

Next we numerically solve x^* for any combination of a and n from equation (9), plug these values of a, n and x^* into the two equations above and check their sign for a range of parameter values we are interested in. In both cases, all combinations in the range $1 \leq a \leq 1000$ and $1 \leq n \leq 1000$ resulted in negative values for both criteria. Therefore, the results with the mating function $f_2(z)$ are evolutionarily stable and convergence stable in this (very large) parameter range.

6) Derivation and stability analysis of equation (10)

The function $f_3(z) = \min(a\frac{z}{1-z}, 1)$ is continuous, but not differentiable at $z = \frac{1}{1+a}$, as can be seen in this example with a=2:

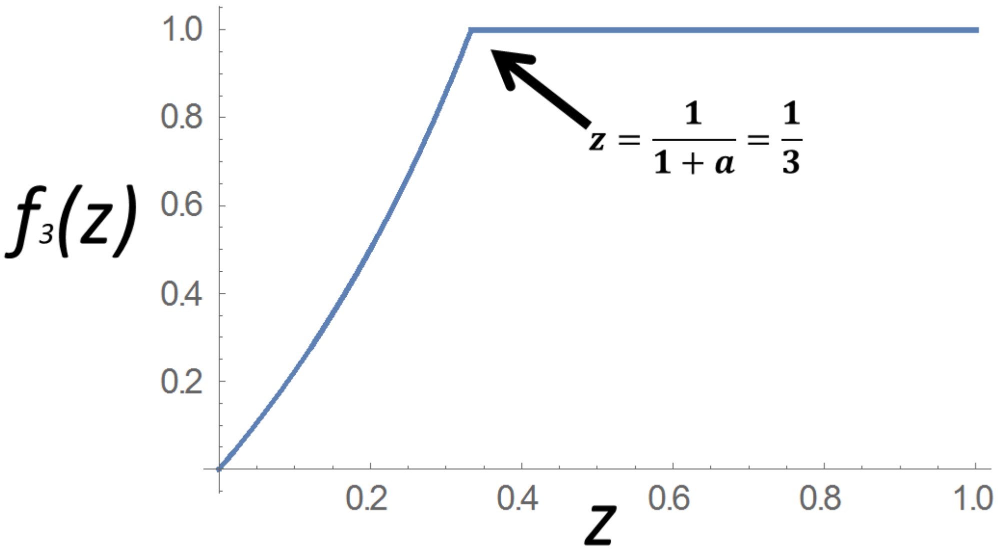

The piecewise nature of this function complicates the analysis in some ways, and the resulting ESS is also piecewise defined.

Firstly, we know from classic LMC theory that when all females are fertilized, the ESS is $x^* = \frac{n-1}{2n}$ (Hamilton 1967; West 2009). This will be the case if $\frac{n-1}{2n} \geq \frac{1}{1+a}$, and we do not need to check the stability of this well-known solution.

However, if $\frac{n-1}{2n} < \frac{1}{1+a}$, we seek the ESS with $f_3(x) = a\frac{z}{1-z}$. Plugging this into equation (4) (main text), we find that $\frac{dw}{dx}\Big|_{x=y=x^*} = 0$ when $x^* = \frac{n+1}{2n}$. However, this solution is only relevant to us if $\frac{n+1}{2n}$ falls in the interval $[0, \frac{1}{1+a}]$ (see figure above). It is easy to check that this can only be true if $a < \frac{n-1}{n+1} < 1$. Such scenarios where a single male cannot fertilize even one female are not realistic for our purposes, and hence the solution $x^* = \frac{n+1}{2n}$ is in itself not

relevant for the purposes of this article.

Instead, whenever fertilization is incomplete, selection drives the sex ratio upwards until all females are fertilized, which happens at $x^* = \frac{1}{1+a}$. If $\frac{n-1}{2n} < \frac{1}{1+a}$, selection ceases here, and if $\frac{n-1}{2n} > \frac{1}{1+a}$, selection continues until $x^* = \frac{n-1}{2n}$ is reached.

Now it still remains to be formally checked whether the candidate ESS $x^* = \frac{1}{1+a}$ is stable when $\frac{n-1}{2n} < \frac{1}{1+a}$. Fitness is not differentiable at this point, but instead of a second derivative analysis, we can show that $\left.\frac{dw}{dx}\right|_{y=x^*}$ changes sign from + to – as x passes $x^* = \frac{1}{1+a}$. This shows that deviant mutants cannot invade, and hence $x^* = \frac{1}{1+a}$ is an ESS.

To check this, we need only compute $\frac{dw}{dx}$ for the left side of $x = x^* = \frac{1}{1+a}$. This is because the right side is in the regime of standard LMC. We assume $\frac{n-1}{2n} < \frac{1}{1+a}$, and therefore in this region we already know that selection and the derivative are negative.

For the left side we use $f_3(x) = a\frac{z}{1-z}$ and find $\frac{dw}{dx} =$ $\frac{a(2(x-y)^2+n(-1+4x-4y)(-1+y)+n^2(1-3y+2y^2))}{(x+n(-1+y)-y)^2}$, which can be shown to be positive when $x < \frac{1}{1+a}$ and $y = x^* = \frac{1}{1+a} < \frac{1}{2}$.

For convergence stability, we must show that $\left.\frac{dw}{dx}\right|_{x=y}$ similarly changes sign from + to – as y passes $x^* = \frac{1}{1+a}$. This shows that if the population is perturbed from the ESS, it will return to

it, and hence $x^* = \frac{1}{1+a}$ is convergence stable.

Again, with similar justification as above, we need only check the left side; on the right side, the derivative is known to be negative due to the stability of the standard LMC result.

For the left side we find that $\left.\frac{dw}{dx}\right|_{x=y} = \frac{a(1+n-2ny)}{n-ny} = a\frac{\frac{1}{n}+1-2y}{1-y}$. Now, because $y < x^* = \frac{1}{1+a} \leq \frac{1}{2}$, both the numerator and denominator are necessarily positive, and hence the derivative is positive.

Therefore equation (7) in the main text is evolutionarily stable and convergence stable.

References for supplementary material

Abe, J., Y. Kamimura, N. Kondo, and M. Shimada. 2003. Extremely female-biased sex ratio and lethal male-male combat in a parasitoid wasp. Behav. Ecol. 14:6–11.

Borsa, P., and F. Kjellberg. 1996. Secondary sex ratio adjustment in a pseudo-arrhenotokous insect, *Hypothenemus hampei* (Coleoptera: Scolytidae). C. R. Acad. Sci. Paris 319:1159–1166.

Cooperband, M. F., R. W. Matthews, and S. B. Vinson. 2003. Factors affecting the reproductive biology of *Melittobia digitata* and failure to meet the sex ratio predictions of Hamilton's local mate competition theory. Entomol. Exp. Appl. 109:1–12.

Cremer, S., and J. Heinze. 2002. Adaptive production of fighter males: queens of the ant *Cardiocondyla* adjust the sex ratio under local mate competition. Proc. R. Soc. Lond. B 269:417–422.

Eshel, I., U. Motro, and E. Sansone. 1997. Continuous stability and evolutionary convergence. Journal of Theoretical Biology 185:333-343.

Frank. 1985. Hierarchical selection theory and sex ratios. II. On applying the theory, and a test with fig wasps. Evolution 39: 949–964.

Hamilton, W. D. 1967. Extraordinary sex ratios. Science 156:477-488.

Herre, E. A. 1985. Sex ratio adjustment in fig wasps. Science 228: 896–898.

Jackson, D. J. 1966. Observations on the biology of *Caraphractus cinctus* Walker (Hymenoptera: Mymaridae), a parasite of the eggs of Dytiscidae (Coleoptera) III. The adult life and sex ratio. Trans. R. Ent. Soc. 118:23–49.

King, B. H., and S. E. Seidl. 1993. Sex-ratio response of the parasitoid wasp *Muscidifurax raptor* to other females. Oecologia 94:428–433.

King, B. H., and S. W. Skinner. 1991a. Proximal mechanisms of the sex ratio and clutch size responses of the wasp *Nasonia vitripennis* to parasitized hosts. Anim. Behav. 42:23–32.

King, B. H. 1989. A test of local mate competition theory with a solitary species of parasitoid wasp, *Spalangia cameroni*. Oikos 55:50–54.

Luck, R. F., J. A. M. Janssen, J. D. Pinto, and E. R. Oatman. 2001. Precise sex allocation, local mate competition, and sex ratio

shifts in the parasitoid wasp *Trichogramma pretiosum*. Behav. Ecol. Sociobiol. 49:311–321.

Molbo, D., and E. D. Parker. 1996. Mating structure and sex ratio variation in a natural population of *Nasonia vitripennis*. Proc. R. Soc. Lond. B 263:1703–1709.

Moore, J. C., S. G. Compton, M. J. Hatcher, and A. M. Dunn. 2002. Quantitative tests of sex ratio models in a pollinating fig wasp. Anim. Behav. 64:23–32.

Nagelkerke, C. J., and M. W. Sabelis. 1996. Hierarchical levels of spatial structure and their consequences for the evolution of sex allocation in mites and other arthropods. Am. Nat. 148:16–39.

Peer, K., and M. Taborsky. 2004. Female ambrosia beetles adjust their offspring sex ratio according to outbreeding opportunities for their sons. J. Evol. Biol. 17:257–264.

Rabinovich, J. E., M. T. Jorda, and C. Bernstein. 2000. Local mate competition and precise sex ratios in *Telenomus fariai* (Hymenoptera: Scelionidae), a parasitoid of triatomine eggs. Behav. Ecol. Sociobiol. 48:308–315.

Roeder, C. 1992. Sex ratio response of the two-spotted spider mite (*Tetranychus urticae* Koch) to changes in density under local mate competition. Can. J. Zool. 70:1965–1967.

Roeder, C., R. Harmsen, and S. Mouldey. 1996. The effects of relatedness on progeny sex ratio in spider mites. J. Evol. Biol. 9:143–151.

Sagarra, L. A., C. Vincent, and R. K. Stewart. 2000. Mutual interference among female *Anagyrus kamali* Moursi (Hymenoptera: Encyrtidae) and its impact on fecundity, progeny production and sex ratio. Biocontrol Sci. Techn. 10:239–244.

Salt, G. 1936. Experimental studies in insect parasitism. IV. The effect of superparasitism on populations of *Trichogramma evanescens*. J. Exp. Biol. 13:363–375.

Schwartz, A., and D. Gerling. 1974. Adult biology of *Telenomus remus* (Hymenoptera: Scelionidae) under laboratory conditions. Entomophaga 19:483–492.

Shuker, D. M., and S. A. West. 2004. Information constraints and the precision of adaptation: sex ratio manipulation in wasps. Proc. Natl. Acad. Sci. U.S.A. 101:10363–10367.

Takagi, M. 1986. The reproductive strategy of the gregarious parasitoid, *Pteromalus puparum* (Hymenoptera: Pteromalidae) 2.

Host size discrimination and regulation of the number and sex ratio of progeny in a single host. Oecologia 70:321–325.

Waage, J. K., and J. A. Lane. 1984. The reproductive strategy of a parasitic wasp II. Sex allocation and local mate competition in *Trichogramma evanescens*. J. Anim. Ecol. 53:417–426.

van Welzen, C. R. L., and J. K. Waage. 1987. Adaptive responses to local mate competition by the parasitoid, *Telenomus remus*. Behav. Ecol. Sociobiol. 21:359–365.

Werren, J. H. 1980. Sex ratio adaptations to local mate competition in a parasitic wasp. Science 208:1157–1159.

———. 1983. Sex ratio evolution under local mate competition in a parasitic wasp. Evolution 37:116–124.

West, S. 2009. Sex Allocation. Princeton University Press.

West, S. A., D. M. Shuker, and B. C. Sheldon. 2005. Sex-ratio adjustment when relatives interact: A test of constraints on adaptation. Evolution 59:1211-1228.
